# A longitudinal study of children’s outside play using family environment and perceived physical environment as predictors

**DOI:** 10.1186/1479-5868-11-76

**Published:** 2014-06-16

**Authors:** Teun Remmers, Suzanne ML Broeren, Carry M Renders, Remy A Hirasing, Amy van Grieken, Hein Raat

**Affiliations:** 1 Department of Public Health, Erasmus MC University Medical Center, P.O. Box 2040, 3000 Rotterdam, CA, The Netherlands; 2 Department of Public and Occupational Health, EMGO Institute of Health and Care Research, VU University Medical Center Amsterdam, Amsterdam, The Netherlands; 3 Institute of Health Sciences, Faculty of Earth and Life Sciences, EMGO Institute of Health and Care Research, VU University Amsterdam, Amsterdam, The Netherlands; 4 Department of Epidemiology, CAPHRI School for Public Health and Primary Care, Maastricht University Medical Center+, Maastricht, The Netherlands

## Abstract

**Background:**

A natural and cheap way of increasing children’s physical activity is stimulating unstructured outside play.

**Purpose:**

This study examined whether characteristics of the family and perceived physical environment were associated with the duration of children’s outside play.

**Methods:**

Parents participating in the “Be Active, Eat Right” cluster RCT control group (*N* = 2007) provided information on potential predictors of outside play (i.e. family and perceived physical environment) of their 5-year-old child by questionnaire. Child outside play was assessed by parental reports both at five and seven years. Linear regression analyses, adjusted for seasonality, were performed to evaluate associations between potential predictors and child outside play. Linear mixed models were fitted to evaluate the relationship between potential predictors and the development of outside play over two years, with season entered as a random factor.

**Results:**

Family environment was the strongest construct predicting child outside play, while parent perceived physical environment had no significant association with child outside play. Parental habit strength and the presence of rules were the strongest predictors of increased outside play. Parent perceived difficulty in improving child outside play was the strongest predictor of decreased outside play.

**Conclusion:**

Family environment predicted child outside play and not perceived physical environment. Parental rules and habit strength regarding improving outside play were associated with an improvement of child’s engagement in outside play.

## Background

A natural and cheap way of increasing children’s physical activity (PA) is by stimulation of outside play, defined as play behavior without any given tasks or goals; unstructured free play. Child outside play (OP), as a specific type of PA behavior, has been shown to increase a child’s total PA level [1,2] and children who spent more time outdoors were shown to be more active than children who spent less time outdoors [3-5]. Recommendations from an expert committee on the prevention of childhood obesity acknowledge unstructured play as most appropriate to increase PA in young children [6]. OP is also positively associated with children’s social skills as they learn to account for other children [7-9] and provides children with opportunities to acquire new motor skills such as climbing, jumping, hanging and sliding in a self-regulatory way [10]. Studies have shown that relatively minor adjustments to school playgrounds lead to increases in PA, which makes OP relatively modifiable [11]. In order to enable effective increasing of child’s engagement in this promising type of PA behavior, determinants of this behavior should be assessed.

Previous studies mainly focused on determinants of PA in general. These studies found that gender (i.e. male), child’s PA enjoyment, and summer and spring season were consistently related with higher levels of PA in children [12]. There may be specific determinants for OP as a specific type of children’s PA behavior, however studies focusing on determinants of OP specifically are scarce. Currently, two studies have specifically examined determinants of OP, also investigating attributes of the physical environment (PE). A cross-sectional study reported that lower parental education and the importance parents ascribe to OP were associated with more OP in 4- to 12-year-old children [13]. A longitudinal study reported that children’s outdoor tendencies and parental encouragement were related to increases in OP across a time span of five years [14]. Both studies indicate that family environment may play an important role in child’s duration of OP.

Studies that incorporate a longitudinal design and use a broader range of environmental variables (e.g., parent perceived safety) to identify determinants of OP are warranted [14]. Therefore, the present study examined whether the family environment and perceived PE are associated with the duration of children’s OP behavior, acknowledging the potential role of gender and seasonal variety and using a broad range of variables covering both the family environment and perceived PE.

## Methods

### Study design and population

The present study was embedded in the “Be Active, Eat Right” study, a cluster RCT investigating the effect of an overweight prevention protocol described in detail elsewhere [9]. The Medical Ethics Committee of the Erasmus University Rotterdam Medical Center approved the study protocol (reference number MEC-2007-163). All parents provided written informed consent.

This study used a longitudinal design to evaluate determinants of OP development over time. We limited our sample to participants that were allocated to youth healthcare teams participating in the control condition. In the control condition, all parents (N = 3,942) were requested to fill in and return a baseline questionnaire at enrollment (child age five), which assessed socio-demographic characteristics, family environment and parent-perceived PE and the duration of OP (2007–2010). Specific variables of family environment were based on established theories, such as social cognitive theory (e.g., self-efficacy) [15], theory of planned behavior (e.g., intention, attitudes) [16], and social leaning theory (e.g., modeling of spouses and parents) [17]. Variables of perceived PE were based on promising results of earlier studies evaluating the influence of the environment on children’s health behavior (e.g., presence of sidewalks, parent perceived safety) [18].

At age seven, duration of OP was assessed again by questionnaire. Records with missing data on the outcomes of interest (i.e. OP at age five and seven, n = 1,895) were excluded from the analyses. By doing so, the population for analysis consisted of N = 2,007 parents and children.

### Measurements

#### Socio-demographic characteristics

The child’s gender, age (in years) and ethnic background were assessed. Child’s ethnic background was considered to be “non-Dutch” when at least one of the parents was born abroad, as defined by Statistics Netherlands. Height and weight of the child were measured at age five by healthcare professionals using standardized protocols [19].

Respondents were either the father or the mother of the child, and parental gender was entered as a potential confounder in subsequent analyses. From this point onwards, respondent will be described as “parent.” Parental age and parental education (low, mid-low, mid-high, high) were assessed in the baseline questionnaire [20].

#### Family environment and physical environment

A full description of constructs, construct properties, items and response scales is presented in the Additional file 1: Table S1.

#### Child outside play

OP was defined by the total duration of unstructured OP of the child in an average week. Note that this is without organized sports, school PE and/or active transport. At both baseline (child age five) and two-year follow-up (child age seven), OP was assessed using an identical set of questions. Total duration of OP was therefore computed in a similar way for both time points, as presented below.

First, parents were asked how many weekdays and weekend days in an average week their child played outside. Second, parents were asked to indicate the average duration in the morning, noon and evening that their child played outside, again separately for weekdays and weekend days. We computed total minutes of OP in the morning, noon and evening. Responses were multiplied by the indicated number of days that the child played outside, separately for weekdays and weekend days. Finally, we summed weekdays and weekend days in order to arrive at the average minutes of OP. We used the date on which the questionnaire was completed to classify the season (i.e. winter, spring, summer and autumn) of both OP at age five and seven. Season was then used as a confounder in all subsequent analyses.

#### Statistical analysis

Characteristics of the population for analysis at child age five were evaluated with descriptive statistics. As PA levels have shown to considerably differ between boys and girls [21], we explored this potential gender difference with chi-square tests and t-tests for categorical and continuous levels of measurements, respectively. Based on the results described in Table 1, we decided not to stratify our subsequent analyses for child gender, as no significant differences were found for the predictors.

**Table 1:** Baseline characteristics at child age five years

	**Total (*n*** **= 2007)**	**Boys (*n*** **= 1013)**	**Girls (*n *=994)**
**Child socio-demographics**			
Age at baseline; mean years (sd) (missing *n* = 6)	5.75 (0.42)	**5.77 (0.42)**	**5.72 (0.41)**
Ethnic background; *n* Dutch (%) (missing *n* = 27)	1804 (91.10)	996 (98.61)	974 (98.38)
BMI; mean kg/m^2^ (sd) (missing *n* = 6)	15.39 (1.47)	15.42 (1.35)	15.36 (1.59)
Average minutes of outside play; mean (sd) (missing *n* =0)	108.99 (65.01)	**112.72 (65.86)**	**104.72 (63.41)**
Outside play ≥ 60 minutes per day, *n* (%) (missing =0)	76.3	78.9	73.6
**Parental socio-demographics**			
Age; mean years (sd), in years (missing *n* = 6)	37.14 (4.38)	37.07 (4.37)	37.21 (4.40)
Gender; *n* male (%) (missing *n* = 7)	179 (9.00)	94 (9.31)	85 (8.59)
Ethnic background; *n* Dutch (%) (missing *n* = 1)	1909 (95.20)	996 (97.67)	943 (96.38)
Education level; *n* (%) (missing *n* = 1)			
Low	50 (2.50)	23 (2.27)	27 (2.72)
Mid-low	288 (14.40)	142 (14.03)	146 (14.69)
Mid-high	902 (45.00)	463 (45.75)	439 (44.16)
High	766 (38.20)	384 (37.94)	382 (38.43)
BMI; mean kg/m^2^ (sd) (missing *n* = 32)	23.94 (7.98)	24.11 (10.65)	23.77 (3.60)
**Family environment**			
Parental attitude; meanscore (sd) (missing *n* = 16)	3.61 (1.05)	3.60 (1.06)	3.62 (1.04)
Family attitude; meanscore (sd) (missing *n* = 42)	2.76 (1.04)	2.73 (1.04)	2.79 (1.04)
Self-confidence; *n* agree (%) (missing *n* = 86)	1021 (53.10)	529 (54.37)	492 (51.90)
Perceived difficulty; *n* agree (%) (missing *n* = 53)	220 (11.30)	107 (10.81)	113 (11.72)
Habit strength; *n* agree (%) (missing *n* = 30)	1607 (81.30)	**836 (83.52)**	**771 (79.00)**
Intention to improve; *n* agree (%) (missing *n* = 51)	881 (45.00)	438 (44.20)	443 (45.91)
Monitoring; *n* frequent (%) (missing *n* = 7)	1565 (78.30)	794 (78.69)	711 (77.80)
Active encouragement; *n* frequent (%) (missing *n* = 12)	1639 (82.60)	822 (82.28)	817 (82.86)
Child autonomy; *n* frequent (%) (missing *n* = 18)	421 (21.10)	215 (21.39)	206 (20.81)
Perception of outside play; *n* more (%) (missing *n* = 42)	539 (27.40)	**295 (29.65)**	**244 (25.15)**
Presence of rules; % with rules (missing *n* = 118)	814 (43.10)	425 (44.69)	389 (41.47)
Modelling parent, mean days PA (sd) (missing *n* = 169)	4.49 (2.10)	4.49 (2.12)	4.49 (2.08)
Modelling partner, mean days PA (sd) (missing *n* = 157)	4.83 (2.27)	4.83 (2.30)	4.81 (2.24)
Modelling siblings, mean days PA (sd) (missing *n* = 266)	6.58 (1.61)	6.59 (1.62)	6.56 (1.61)
**Perceived physical environment**			
Traffic business; *n* agree (%) (missing *n* = 2)	567 (28.30)	279 (27.57)	288 (29.00)
Safety perception during daytime; *n* agree (%) (missing *n* = 11)	1460 (73.10)	751 (74.50)	709 (71.76)
Safety perception during evenings; *n* agree (%) (missing *n* = 17)	1035 (52.00)	**543 (54.19)**	**492 (49.80)**
Presence of sidewalks; *n* agree (%) (missing *n* = 11)	1365 (68.40)	701 (69.47)	664 (67.27)
Friendliness for children; *n* agree (%) (missing *n* = 10)	1661 (83.20)	847 (84.11)	814 (82.22)
Attractiveness for children; *n* agree (%) (missing *n* = 14)	1502 (75.20)	763 (75.84)	739 (74.50)
Opportunities for outside play; *n* agree (%) (missing *n* = 16)	1390 (69.70)	703 (69.81)	687 (69.60)
Safety without supervision; *n* agree (%) (missing *n* = 12)	1093 (54.80)	557 (55.42)	537 (54.09)

Chi-square for categorical variables and t-test for continuous variables, Numbers printed in **bold** represent *p <* 0.05.

We fitted general linear regression models with predictors at child age five as independent variables and OP of the child at age five as the dependent variable. First, we tested univariate associations between each predictor and OP. Relevant predictors (p < 0.10) were entered in separate models per category of predictors, namely socio-demographic characteristics (Model 1), family environment (Model 2) and perceived PE (Model 3). In Model 4, all predictors were entered simultaneously. In all models, season at child age five was added as a confounder.

We fitted linear regression models between OP at age seven as the dependent variable and selected predictors at child age five as independent variables. Season at age seven was entered as a confounder. All variables were selected and further entered in the model in a similar way as described for the cross-sectional analyses.

Linear mixed models were used to evaluate the influence of the family and perceived PE on the development of OP over two years. We entered OP at five and seven (additional row for a repeated observation), with season at age five and seven entered as a random effect. To investigate the influence of predictors on the development of OP (i.e. change over time) we computed time in years to follow-up by subtracting child age at follow-up from child age at baseline. Subsequently, we modeled interactions between “time to follow-up” and individual predictors to evaluate whether there were associations between the predictor and OP development between child age five and seven. We again selected these predictors based on univariate associations.

Drop-out analyses were performed by means of t-tests for continuous socio-demographic variables (child age, child BMI and parental BMI) and chi-square tests for categorical variables (child gender, child ethnic background, parental ethnic background and parental educational level). All analyses were performed with SPSS version 20.0 (IBM Corp., NY, USA). Multi-collinearity seemed to be a minor issue, as all individual predictors showed variance inflation factors of < 10 [22].

## Results

### Baseline characteristics

Table 1 shows the characteristics of the study population at baseline. Of the children, 50.5% were boys and 49.5% were girls. Children were predominantly of Dutch ethnic background (91.1%). Boys were older than girls and engaged in more minutes of OP than girls. In total, 76.3% of the children engaged in 60 or more minutes of OP per day (i.e. met the WHO guideline of PA). Respondents were predominantly mothers (91.0%), and 95.2% were of Dutch ethnic background. Regarding family environment of the participating children, only 11.3% perceived improving their OP as difficult. Regarding perceived PE, parents of boys perceived more safety in the evening compared to parents of girls.

Compared to children with missing data, the population for analysis consisted of a higher percentage of children with a Dutch ethnic background, a higher percentage of parents with a Dutch ethnic background and a higher percentage of high education parents (all p < 0.05). This means that relatively lower educated parents were more likely to have one or more missing values on their OP.

### Associates of OP at child age five

Table 2 shows the cross-sectional associations between predictors at child age five and OP at child age five. Parental self-confidence, traffic business and the presence of sidewalks in the neighborhood showed a non-significant univariate association with OP (p > 0.10) and were therefore not included in the models.

**Table 2:** Linear regression models examining factors related to child outside play at age five (min/day)

	**Model 1**	**Model 2**	**Model 3**	**Model 4**
	**beta (95% CI)**	**beta (95% CI)**	**beta (95% CI)**	**beta (95% CI)**
***n***	**1940**	**1390**	**1968**	**1337**
**Socio-demographics**				
Child age at baseline	**14.42 (6.71 to 22.14)**			**13.61 (5.13 to 22.08)**
Child gender; boy	**6.52 (1.12 to 11.91)**			4.07 (−1.79 to 9.93)
Child ethnic background; Dutch	1.79 (−11.99 to 15.57)			−4.45 (−20.26 to 11.36)
Child BMI	**2.11 (0.25 to 3.96)**			2.00 (−0.22 to 4.21)
Parental age at baseline	−0.62 (−1.28 to 0.03)			−0.33 (−1.08 to 0.42)
Parental ethnic background; Dutch	**22.94 (4.74 to 41.14)**			11.34 (−11.12 to 33.80)
Parental BMI	0.26 (−0.08 to 0.60)			**1.14 (0.26 to 2.02)**
Parental education level				
Low	reference			reference
Mid-low	15.61 (−7.34 to 38.55)			15.64 (−21.27 to 52.56)
Mid-high	7.82 (−14.40 to 30.04)			10.35 (−25.97 to 46.67)
High	−3.90 (−26.26 to 18.46)			−2.04 (−38.45 to 34.37)
**Family environment**^**1**^				
Parental attitude (agree)		**−4.32 (−8.01 to −0.63)**		**−3.78 (−7.46 to −0.10)**
Family attitude (agree)		−2.30 (−6.15 to 1.55)		−2.41 (−6.27 to 1.46)
Perceived difficulty (agree)		**−18.06 (−28.12 to −8.00)**		**−16.33 (−26.41 to −6.26)**
Habit strength (agree)		**33.89 (25.52 to 42.75)**		**33.41 (25.05 to 41.77)**
Intention to improve (agree)		**−10.81 (−17.64 to −3.98)**		**−12.83 (−19.73 to −5.92)**
Presence of rules		**23.98 (17.60 to 30.36)**		**19.87 (13.44 to 26.30)**
Presence of monitoring		4.90 (−2.90 to 12.70)		4.87 (−2.97 to 12.71)
Presence of active encouragement		−5.26 (−12.71 to 2.19)		−3.00 (−10.48 to 4.48)
Child autonomy (agree)		**12.16 (3.83 to 20.49)**		**11.66 (3.30 to 20.02)**
Modelling parent (#days physically active)		0.88 (−0.63 to 2.39)		0.58 (−0.95 to 2.10)
Modelling partner (#days physically active)		0.39 (−0.99 to 1.78)		0.84 (−0.58 to 2.25)
Modelling siblings (#days physically active)		**5.01 (3.02 to 6.99)**		**4.82 (2.81 to 6.83)**
**Perceived physical environment**^**1**^				
Safety perception during daytime (agree)			−0.81 (−9.67 to 8.05)	0.20 (−9.54 to 9.94)
Safety perception during evenings (agree)			**11.82 (4.89 to 18.74)**	5.21 (−2.15 to 12.58)
Friendliness for children (agree)			4.43 (−6.52 to 15.37)	−2.63 (−14.53 to 9.27)
Attractiveness for children (agree)			0.84 (−9.03 to 10.72)	−2.79 (−13.43 to 7.85)
Safety of outside play without supervision (agree)			1.91 (−4.99 to 8.81)	−0.40 (−7.89 to 7.09)
R-square^2^	0.13	0.27	0.10	0.31

^1^For details on the measures used see Additional file 1: Table S1.

^2^R square statistic represents the level of variance explained by the general linear model.

Note: numbers printed **bold** represent a statistically significant (*p <* .05) association between the predictor and child outside play at five years old.

The socio-demographic variables explained 13% of the variance in OP. Two variables were consistently related to OP: child age and child BMI. Specifically, a one-year increment in child’s age at baseline was associated with a 13.56-minute increase in OP per day.

Family environment variables explained 27% of the variance in OP at age five. Parents who indicated difficulty towards improving OP reported their child to play outside 16.33 (95% CI = −26.41 to −6.26) minutes less per day. Parents with a habit towards improving OP reported 33.41 (95% CI = 25.05 to 41.77) more minutes of OP. The presence of rules regarding OP was associated with 19.87 (95% CI = 13.44 to 26.30) more minutes of OP. Parents of children that indicated intention to improve their child’s OP reported 12.83 (95% CI = −19.73 to −5.92) less minutes of OP.

Perceived PE together explained 10% of the variance in OP at age five. No variables within perceived PE were significantly related with OP at child age five.

### Associates of OP at child age seven

Table 3 shows longitudinal associations between predictors at child age five and OP at child age seven. Child and parental ethnic background, parental BMI, parental self-confidence, traffic business in the neighborhood and the presence of sidewalks in the neighborhood showed a non-significant univariate association with OP (p > 0.10) and were therefore not included in the models.

**Table 3:** Linear regression models examining factors related to child outside play at age seven (min/day)

	**Model 1**	**Model 2**	**Model 3**	**Model 4**
	**beta (95%CI)**	**beta (95%CI)**	**beta (95%CI)**	**beta (95%CI)**
***n***	**1994**	**1390**	**1968**	**1363**
**Socio-demographics**				
Child age at baseline	**7.98 (0.88 to 15.09)**			4.65 (−3.50 to 12.79)
Child gender; boy	**8.51 (2.68 to 14.34)**			6.36 (−0.20 to 12.93)
Child BMI	1.21 (−0.79 to 3.21)			1.72 (−0.72 to 4.16**)**
Parental age at baseline	−0.85 (−1.55 to −0.15)			−0.41 (−1.23 to 0.41)
Parental education level				
Low	reference			reference
Mid-low	17.59 (−2.34 to 37.52)			8.87 (−19.62 to 37.35)
Mid-high	−1.73 (−20.66 to 17.20)			−8.25 (−35.67 to 19.17)
High	**−19.50 (−38.58 to −0.41)**			**−26.63 (−54.10 to 0.84)**
**Family environment**^**1**^				
Parental attitude (agree)		−3.80 (−7.97 to 0.37)		−3.97 (−8.08 to 0.15)
Family attitude (agree)		−3.22 (−7.57 to 1.13)		−2.87 (−7.19 to 1.45)
Perceived difficulty (agree)		**−24.44 (−35.81 to −13.06)**		**−22.11 (−33.41 to −10.81)**
Habit strength (agree)		**25.19 (15.72 to 34.67)**		**23.99 (14.61 to 33.36)**
Intention to improve (agree)		−1.12 (−8.84 to 6.60)		−2.88 (−10.57 to 4.81)
Presence of rules		**21.49 (14.28 to 28.69)**		**16.46 (9.26 to 23.67)**
Presence of monitoring		2.97 (−5.83 to 11.78)		5.30 (−3.42 to 14.02)
Presence of active encouragement		**−10.82 (−19.25 to −2.39)**		**−8.91 (−17.33 to −0.48)**
Child autonomy (agree)		5.48 (−3.95 to 14.90)		4.80 (−4.59 to 14.19)
Modelling parent (#days physically active)		0.50 (−1.21 to 2.20)		0.45 (−1.24 to 2.15)
Modelling partner (#days physically active)		1.49 (−0.07 to 3.05)		**1.85 (0.27 to 3.42)**
Modelling siblings (#days physically active)		1.06 (−1.18 to 3.30)		1.27 (−0.99 to 3.52)
**Perceived physical environment**^**1**^				
Safety perception during daytime (agree)			−3.57 (−13.24 to 6.10)	2.88 (−8.03 to 13.79)
Safety perception during evenings (agree)			**8.68 (1.18 to 16.19)**	3.54 (−4.68 to 11.76)
Friendliness for children (agree)			4.63 (−7.31 to 16.57)	−5.57 (−18.66 to 7.72)
Attractiveness for children (agree)			4.88 (−5.89 to 15.64)	3.21 (−8.62 to 15.05)
Safety of outside play without supervision (agree)			3.93 (−3.57 to 11.43)	0.12 (−8.22 to 8.47)
R-square^2^	0.10	0.15	0.06	0.20

^1^For details on the measures used see Additional file 1: Table S1.

^2^R square statistic represents the level of variance explained by the general linear model.

Note: numbers printed **bold** represent a statistically significant (*p <* .05*)* association between the predictor and child outside play at five years old.

Socio-demographic variables explained 10% of the variance in OP at age seven. Only education was consistently related with OP (p < 0.05); parents with high education reported their child to play outside 28.40 minutes less (95% CI = −55.66 to −1.14) compared to parents with low education.

Family environment variables together explained 15% of the variance in OP at age seven. Parents who indicated difficulty towards improving OP reported 22.11 (95% CI = −33.41 to −10.81) less minutes of OP. Parents with a habit towards improving OP reported 23.99 (95% CI = 14.61 to 33.61) more minutes of OP. The presence of rules regarding OP was associated with 16.46 (95% CI = 9.26 to 23.67) more minutes of OP. Parental active encouragement at child age five was associated with 8.91 (95% CI = −17.33 to −0.48) less minutes of OP at child age seven.

Perceived PE together explained 6% of the variance in OP at age seven. No variables within perceived PE were significantly related with OP at child age seven.

### Associates of OP development between child age five and seven

Child age, child and parental ethnic background, parental attitude, family attitude, intention to improve, the presence of rules and positive modeling of siblings were included in the models, presented in Table 4, as these variables showed significant univariate associations (i.e. p > 0.10). This means that none of the variables within perceived PE were related to OP (i.e. change between child age five and seven).

**Table 4:** Linear mixed models examining factors related to child outside play development between age five and seven (min/day)

	Adjusted effect* at baseline	Adjusted effect* at 2 years follow-up	***p *** **for time interaction**
***n = *** **1552**			
**Socio-demographics**			
Child age at baseline	**26.17 (19.19 to 33.14)**	5.71 (−2.57 to 13.99)	< 0.01
Child ethnic background; Dutch	−3.03 (−18.47 to 12.40)	−1.22 (−18.73 to 16.28)	0.86
Parental ethnic background; Dutch	**21.28 (0.56 to 42.00)**	8.19 (−15.79 to 32.17)	0.36
**Family environment**^**1**^			
Parental attitude (agree)	**−5.83 (−9.32 to −2.34)**	**−5.74 (−9.95 to −1.52)**	0.97
Family attitude (agree)	−5.07 (−8.67 to −1.47)	−3.59 (−7.88 to 0.70)	0.56
Intention to improve (agree)	**−11.33 (−18.01 to −4.66)**	−4.34 (−12.15 to 3.46)	0.14
Presence of rules	**29.71 (23.89 to 35.54)**	**23.85 (17.04 to 30.66)**	0.15
Modelling siblings (#days physically active)	**7.56 (5.76 to 9.36)**	**3.95 (1.87 to 6.03)**	< 0.01

^1^For details on the measures used see Additional file 1: Table S1, *Adjusted for season, age at baseline and follow-up. Adjusted effects are not exactly equal to results in Tables 2 and 3 because of differences in included covariates, and model specifications.

Note: numbers printed **bold** represent statistically significant associations between the predictor and child outside play.

Regarding socio-demographic variables, child age at baseline was related to a significant change in OP between baseline and follow-up (Table 4; p < 0.05). This was possible as all children were not precisely five years old at baseline. This means that a higher child age was related to higher engagement in OP at baseline, but that this attenuated significantly over time.

Regarding family environment, the intention to improve OP and the presence of rules were both associated with a decrease of OP over time compared to baseline (i.e. Table 2). This means that modeling of sibling was related to higher engagement in OP at baseline, but that this attenuated significantly over time.

## Discussion

This study has shown that family environment was the strongest construct of variables predicting OP, compared to socio-demographic characteristics and perceived PE. More specifically, this study has demonstrated that habit strength and the presence of rules were positive, strong and stable predictors of OP over time. Positive modeling by siblings and child age at baseline were also positive predictors. In contrast, parent perceived difficulty in improving OP was a negative (i.e. related to less minutes of OP), relatively strong and stable predictor of OP over time.

As this study focused on unstructured OP, results are not generalizable to organized sports, school PE and/or active transport PA. Therefore, one should be cautious in directly relating an increase in OP with more PA energy expenditure (PAEE) and the accompanied benefits regarding childhood obesity. However, considering the various benefits of OP (e.g., increased PA, motor abilities and social skills), it may contribute to an improved general health status of the child over time.

This study has found no relationship between perceived PE and OP, which is in line with a cross-sectional study among 4- to 12-year-old children [13] and a longitudinal study among 5- to 6-year-old children [14]. Hypothetically, an explanation for this may be that parents have other perceptions regarding PA opportunities in their neighborhood than their children, which may also explain the relatively weak associations regarding attractiveness, child friendliness and traffic busyness. Therefore, future studies are urged to assess both parental and children’s motives for OP. Another explanation may be that attributes of perceived PE are moderated or mediated by family environment. Future studies are encouraged to investigate these mechanisms.

Our study has found that a positive parental attitude and family attitude were related to less time spent on OP. An explanation for this contra-intuitive finding may be that some parents in the present study perceived that their child needed more PA or OP and these parents had the intention to improve but were unable to achieve this yet compared to other children [23]. Another explanation may be that reverse causation played a role here, as parents may consider their OP as sufficient and therefore did not think their child could/should improve on this. In addition, active parental encouragement towards improving OP was related to less OP over time, which may be explained by the possibility that active encouragement conflicts with the self-regulatory character of OP. It may be suggested that facilitation and providing autonomy may be more effective in promoting OP in children.

Our positive association regarding parental rules and duration of OP were not in line with the findings of Sallis et al. [22]. Discrepancy in the formulation of rules may explain this as Sallis et al. formulated rules merely related to the discouragement of OP (e.g., do not play rough games), while this study solely asked for the presence of rules regarding OP.

This study has demonstrated a negative association between parent-perceived difficulty in improving their child’s OP and OP at child age five and seven. This indicates that parents are able to indicate difficulties, and that the presence of these difficulties was indeed associated with relatively low levels of OP. Future studies should investigate these difficulties more thoroughly to identify what the exact difficulties are that parents are struggling with (e.g., time constraints, child’s friends to play with, etc.).

### Strengths and weaknesses

A strength of the present study is its longitudinal design, including a relatively large sample. In addition, this study assessed PA through a whole year, subsequently adjusting for the effect of seasonality. The present study’s selective analytical approach resulted in sufficient model stability. This can be seen in the stability of parameters across model-variations (e.g., stability of parental age between models 1 and 4 of Table 2) and the relatively large sample size. In addition, multi-collinearity seemed to be a minor issue, as all individual predictors showed variance inflation factors of < 10 [24].

Our drop-out analyses showed that relatively lower educated parents were more likely to have one or more missing values on their OP. This may be due to misunderstanding the Dutch translation of “OP” or the relative complexity of the question assessing OP. In addition, as we are aware that in some of our cross-sectional results reversed causation may have played a role, we urge future studies to use longitudinal designs in order to unravel this, especially regarding the relationship between parental and family attitude and OP.

To date, OP can only be assessed by parental report, as often-used single objective measurements (e.g., accelerometers or heart-rate monitors) cannot distinguish OP from other types of PA. However, future studies need to assess OP using objective measures. In this regard, special attention should be directed towards combining global positioning system (GPS), GIS, and accelerometers, whose methodologies yield an objective assessment of domain-specific PA [25,26].

The present study used parental perception of their PE while nowadays more detailed objective assessment of the environment is also available: for example, with the use of geographic information systems (GIS) [27]. Studies that directly compared objective and perceived PE suggest that these two concepts are different but interrelated [18,28,29]. Although studies using perceived physical environment have shown relatively weak associations with objective PA [30,31], consistency was higher when PA was also measured by parental reports [18]. This is supported by several conceptual frameworks, which postulate that perceived PE may be a more proximal function of the objective environment [28,29], as the influence of objective PE is moderated by personal factors and selective daily mobility [32,33]. Therefore, future studies need to include both objective and subjective measures of PE to unravel these phenomena.

Regarding the relative contribution of OP to total PA, only one study estimated the proportion of moderately to vigorously intense PA (MVPA) during OP in special playgrounds adapted to promote PA, and reported that approximately 35% of the time spent in OP was MVPA [34]. Future studies are therefore also encouraged to quantify the contribution of OP to total physical activity energy expenditure. Irrespective of the intensity of OP, approximately 76% of the children engaged in ≥ 60 minutes of OP. As this may be higher than other studies, the present results may be limited in their generalizability.

## Conclusion

Although other studies reported that the attributes of perceived PE are associated with a child’s PA behavior, this study has revealed that family environment overpowers these attributes of perceived PE at child age five and seven. This means that a supportive family environment is the key determinant of regular OP. Future studies are encouraged to investigate the potential moderating role of family environment in the relationship between perceived PE and OP.

More specifically, this study has demonstrated that the presence of parental rules and parental habit strength was associated with more OP, and perceived difficulty was associated with less OP. As these relationships were stable over two years, future interventions to increase OP should parents to set clear rules about OP and subsequently foster habit formation regarding OP. Future studies should also implement qualitative methods to investigate reasons behind, among others, the parental perception of difficulty to improve OP. These studies may provide interesting insights for the development of evidence-based intervention programs supporting parents to promote OP.

## Competing interests

All authors declare that they have no competing interests.

## Authors’ contributions

HR, RH, and CR originated the idea for the study and its design and were responsible for acquiring the grant for the study. AG acquired the data. TR drafted the manuscript and performed the statistical analyses. SB and AG critically reviewed the statistical analyses and interpretation. All authors critically read and approved the final manuscript.

## Supplementary Material

Additional file 1: Table S1 Items assessing family and physical environment with regard to child outside play engagement. Click here for file
