# Supplementary material for: A longitudinal study of children’s outside play using family environment and perceived physical environment as predictors
Source: Int J Behav Nutr Phys Act. 2014 Jun 16;11:76. doi: 10.1186/1479-5868-11-76 (PMC4075777; doi:10.1186/1479-5868-11-76)
Supplement: Additional file 1: Table S1 — Items assessing family and physical environment with regard to child outside play engagement. [file 1479-5868-11-76-S1.doc]

**Additional file 1: Table S1: Items assessing family and physical environment with regard to child outside play engagement**

| Construct | Construct properties | Construct description | Translated item | Response scale | Dichotomous scale |
| --- | --- | --- | --- | --- | --- |
| **Family environment** | |  |  |  |  |
| Parental attitude | Cronbach’s alpha 0.86 | Parental attitude to improve child engagement in outside play | I think it is important for my child to improve child engagement in outside play | 1=totally disagree to 5= totally agree | - |
|  | Scale range 1-5 |  | I think it is good for my child to improve child engagement in outside play | 1=totally disagree to 5=totally agree | - |
|  |  |  | I think it is healthy for my child to improve child engagement in outside play | 1=totally disagree to 5=totally agree | - |
| Family attitude | Cronbach’s alpha 0.85 | Family attitude to improve child engagement in outside play | I think my child should increase engagement in outside play | 1=totally disagree to 5=totally agree | - |
|  | Scale range 1-5 |  | My partner thinks our child should increase engagement in outside play | 1=totally disagree to 5=totally agree | - |
|  |  |  | My family thinks my child should increase engagement in outside play | 1=totally disagree to 5=totally agree | - |
| Perceived difficulty | - | Parental perception of their difficulties in improving child engagement in outside play | It is difficult to let my child engage in more outside play | 1=totally agree to 5=totally disagree | 1=agree (strongly agree, agree), 0=not agree (not agree/not disagree, disagree, strongly disagree) |
| Self confidence | - | Parental confidence in their own ability to improve child engagement in outside play | I can let the child engage in more outside play | 1=totally agree to 5=totally disagree | 1=agree (strongly agree, agree), 0=not agree (not agree/not disagree, disagree, strongly disagree) |
| Intention to improve | - | Parental intention to improve child engagement in outside play | I am planning to improve my child’s engagement in outside play | 1=totally disagree to 5=totally agree | 1=agree (strongly agree, agree), 0=not agree (not agree/not disagree, disagree, strongly disagree) |
| Habit strength | - | Parental perception improving child engagement in outside play is a habit for them | It is a habit for me to let my child engage in more time of outside play | 1=totally disagree to 5=totally agree | 1=agree (strongly agree, agree), 0=not agree (not agree/not disagree, disagree, strongly disagree) |
| Monitoring | - | Parental monitoring of child engagement in outside play | To what extent do you monitor your child’s engagement in outside play? | 1=never to 5=always | 1=frequent (often, always), 0=not frequent (sometimes, seldom, never) |
| Child autonomy | - | Parental control towards child outside play | How often can your child decide for themselves to play outside? | 1=never to 5=always | 1=frequent (often, always), 0=not frequent (sometimes, seldom, never) |
| Active encouragement | - | Degree of parental encouragement towards their child’s outside play | How often do you tell your child to play outside? | 1=never to 5=always | 1=frequent (often, always), 0=not frequent (sometimes, seldom, never) |
| Modeling parent | - | Physical activity behavior of the parent | How many days per week are you physically active? | 0 to 7 | - |
| Modeling partner | - | Physical activity behavior of the partner from the parent | How many days per week is your partner physically active? | 0 to 7 | - |
| Modeling siblings | - | Physical activity behavior of siblings | How many days per week are siblings physically active? | 0 to 7 | - |
| Rules |  | Presence of rules parents report with regard to child outside play | Do you have rules in your home about your child’s outside play? | 1=yes, 0=no | 1=yes, 0=no |
| **Physical environment** | |  |  |  |  |
| Traffic business | - | Parent perceived presence of traffic | In the neighborhood of our family there is a lot of traffic | 1=totally disagree to 5=totally agree | 1=agree (strongly agree, agree), 0=not agree (not agree/not disagree, disagree, strongly disagree) |
| Safety perception during daytime | - | Parent perceived safety regarding outside play during daytime | In the neighborhood of our family it is safe for children to play during the daytime | 1=totally disagree to 5=totally agree | 1=agree (strongly agree, agree), 0=not agree (not agree/not disagree, disagree, strongly disagree) |
| Safety during the evening | - | Parent perceived safety regarding outside play in the evening | In the neighborhood of our family it is safe for children to play in the evening | 1=totally disagree to 5=totally agree | 1=agree (strongly agree, agree), 0=not agree (not agree/not disagree, disagree, strongly disagree) |
| Presence of sidewalks | - | Parent perceived presence of sidewalks for their child to play on | In the neighborhood of our family, the majority of the streets have sidewalks to play on | 1=totally disagree to 5=totally agree | 1=agree (strongly agree, agree), 0=not agree (not agree/not disagree, disagree, strongly disagree) |
| Friendliness for children | - | Parent perceived child-friendliness of their neighborhood | The neighborhood of our family is pleasant to reside with children | 1=totally disagree to 5=totally agree | 1=agree (strongly agree, agree), 0=not agree (not agree/not disagree, disagree, strongly disagree) |
| Attractiveness for children | - | Parent perceived attractiveness of their neighborhood for families with children | The neighborhood of our family is attractive for families with children | 1=totally disagree to 5=totally agree | 1=agree (strongly agree, agree), 0=not agree (not agree/not disagree, disagree, strongly disagree) |
| Opportunities for outside play | - | Parent perceived opportunities of the neighborhood for their child to play | In the neighborhood of our family there is sufficient opportunities for my child to play | 1=totally disagree to 5=totally agree | 1=agree (strongly agree, agree), 0=not agree (not agree/not disagree, disagree, strongly disagree) |
| Safety of outside play without supervision | - | Parent perceived safety regarding outside play without supervision | In the neighborhood of our family it is safe to play outside without supervision of an adult | 1=totally disagree to 5=totally agree | 1=agree (strongly agree, agree), 0=not agree (not agree/not disagree, disagree, strongly disagree) |
